# Wideband tympanometry for evaluating middle ear function and hearing prognosis after endoscopic type I tympanoplasty

**DOI:** 10.3389/fneur.2025.1682975

**Published:** 2025-12-08

**Authors:** Xiaoqian Ren, Yihang Xu, Yijing Yang, Yueqi Zhang, Kexin Zhang, Qinzhuo Ma, Meiyi Pan, Wei Wang

**Affiliations:** 1 Medical School, Tianjin University, Tianjin, China; 2 Department of Otorhinolaryngology Head and Neck Surgery, Tianjin First Central Hospital, Tianjin, China; 3 Institute of Otolaryngology of Tianjin, Tianjin, China

**Keywords:** wideband tympanometry, type I tympanoplasty, wideband absorbance, air-bone gap, resonance frequency

## Abstract

**Objective:**

To investigate the value of wideband tympanometry (WBT) in assessing middle-ear function after endoscopic type I tympanoplasty, and to analyze postoperative changes in wideband absorbance (WBA), resonance frequency (RF), and air-bone gap (ABG) and their relationships with hearing recovery.

**Methods:**

Forty-four patients (44 ears) with unilateral chronic otitis media who underwent endoscopic type I tympanoplasty and completed postoperative follow-up were retrospectively enrolled as the study group. The contralateral normal ears (44 ears) of the same patients served as the control group. Independent-samples t-tests were used to compare postoperative WBA and RF between the two groups, and paired t-tests were used to compare preoperative and postoperative ABG within the study group. Spearman’s rank correlation analysis was used to assess the relationships between postoperative WBA, RF, and ABG. In addition, postoperative WBA at 1000 Hz was compared between temporalis fascia and tragal perichondrium graft subgroups using independent-samples t-tests.

**Results:**

Compared with controls, the study group showed lower mean WBA across 226–8,000 Hz, with an asymmetric M-shaped pattern. Between-group differences in mean WBA were statistically significant in the mid-low (500–1,000 Hz), mid-high (1000–4,000 Hz), and high (4000–8,000 Hz) bands and were most pronounced in the 1,000–4,000 Hz band, whereas the difference in the low-frequency band (226–500 Hz) did not reach statistical significance. The mean RF was significantly lower in the study group than in the control group (*t* = −2.225, *p* < 0.05) and was negatively correlated with mean postoperative ABG (*r* = −0.439, *p* < 0.05). In the operated ears, postoperative ABG was significantly reduced at all tested frequencies between 250 and 4,000 Hz, and WBA at 1000 Hz was negatively correlated with postoperative ABG at the same frequency (*r* = −0.347, *p* < 0.05).

**Conclusion:**

WBT sensitively reflects changes in the mechanical properties of the middle ear after type I tympanoplasty. Reduced WBA at mid-to-high frequencies together with decreased RF indicates increased tympanic membrane mass and reduced stiffness. The negative correlation between WBA at 1000 Hz and postoperative ABG indicates that WBA at this frequency may serve as an objective predictor of postoperative hearing prognosis, providing a quantitative basis for clinical efficacy evaluation.

## 1 Introduction

Chronic otitis media (COM) is characterized by persistent middle-ear inflammation, recurrent otorrhea, hearing loss, and otalgia. Affected patients frequently present with tympanic membrane perforations (TMP), which typically fail to heal spontaneously because of the chronic inflammatory process (1). Surgical intervention is therefore required to repair the perforated eardrum and protect middle-ear function. Type I tympanoplasty (myringoplasty) involves repair of a TMP without ossicular chain reconstruction (2) and is currently the standard procedure for managing TMPs (3, 4). In recent years, endoscopic tympanoplasty has gained popularity because it offers several advantages over microscopic techniques, including a wider and clearer surgical field, shorter operative time, faster recovery, fewer complications, and favorable hearing outcomes (5, 6).

Variations in the stiffness and thickness of an intact tympanic membrane can significantly affect sound transmission (7). Therefore, reconstructing a tympanic membrane that closely resembles the native eardrum is crucial during tympanoplasty. Wideband tympanometry (WBT), which utilizes probe tones across 226–8,000 Hz (107 frequencies), offers higher sensitivity and specificity than conventional 226-Hz tympanometry, particularly for detecting subtle or complex middle-ear pathologies (8). WBT results are typically expressed as wideband absorbance (WBA) or wideband reflectance (WBR). This study aimed to characterize WBT findings after endoscopic type I tympanoplasty, analyze postoperative changes in WBA and hearing recovery as reflected by the air-bone gap (ABG), and explore factors associated with WBA, in order to provide a basis for improved clinical assessment and treatment.

## 2 Materials and methods

### 2.1 Subjects

This retrospective study included 44 patients (44 ears) with unilateral chronic otitis media who underwent endoscopic type I tympanoplasty at the Department of Otorhinolaryngology-Head and Neck Surgery, Tianjin First Central Hospital, between 2023 and 2024. All patients had complete postoperative follow-up data, including basic medical history, pre- and postoperative pure-tone audiometry results, postoperative otoscopic findings, and postoperative conventional 226-Hz tympanometry and WBT measurements. All postoperative assessments (pure-tone audiometry, otoscopic examination, 226-Hz tympanometry, and WBT) were performed on the same day.

The operated ears of these 44 patients constituted the study group, and their contralateral normal ears served as the control group. Inclusion criteria for the study group included: (1) endoscopic type I tympanoplasty performed; (2) a well-healed tympanic membrane on postoperative otoscopic examination; and (3) intraoperative confirmation of intact middle-ear structures without additional middle-ear pathology. Inclusion criteria for the control group included: (1) no definite history of otologic disease; (2) normal auricle and external auditory canal anatomy, with a patent ear canal and intact tympanic membrane; (3) a type A tympanogram on 226-Hz tympanometry with acoustic reflexes within normal limits; and (4) normal hearing or only mild high-frequency sensorineural hearing loss. Written informed consent was obtained from all participants prior to enrollment. The study was approved by the Ethics Committee of Tianjin First Central Hospital (YC-BY-LC-2023-0020).

### 2.2 Surgical technique

All surgeries were performed under general anesthesia using a flap elevation approach. When temporalis fascia was selected as the graft material, a transverse incision approximately 3 cm in length was made along the hairline posterior to the operative ear to harvest the fascia, which was then preserved for later use. When tragal perichondrium was chosen, an incision was made along the free edge of the tragus to harvest the perichondrium, which was subsequently dried and stored. Under endoscopic visualization, a graft bed was prepared medial to the perforation margin. A posteriorly based U-shaped canal skin flap was elevated from the posterior and inferior canal wall, 5 mm to 1 cm lateral to the annulus. The flap was raised to enter the middle ear, which was then explored. Gelfoam was placed in the middle-ear cavity, and the tympanic membrane was reconstructed using the underlay technique, positioning the graft anteriorly under the malleus handle and medial to the canal skin flap. The canal flap was repositioned, and the external auditory canal was packed with Gelfoam and iodoform gauze.

### 2.3 Audiological testing

#### 2.3.1 Tympanometry

The external auditory canal was first inspected to ensure patency and absence of cerumen or other obstruction. Conventional 226-Hz tympanometry was then performed using a GSI TympStar Pro middle-ear analyzer. An appropriate sized probe tip was selected to achieve a hermetic seal. Tympanometric measurements were obtained by sweeping the ear-canal pressure from +200 to −400 daPa to obtain the tympanogram.

#### 2.3.2 Wideband tympanometry

WBT was performed using the wideband module of the Interacoustics Titan IMP440 system. The probe stimulus ranged from 226 to 8,000 Hz. Participants were instructed to swallow immediately before each measurement to equilibrate middle-ear pressure and minimize the effect of negative pressure on WBA. The testing procedure was similar to that used for 226-Hz tympanometry. The instrument recorded and analyzed WBA and RF across the stimulus range, with primary analyses based on WBA at peak pressure.

#### 2.3.3 Pure-tone audiometry

Pure-tone audiometry was performed in a sound-treated booth using GSI-61 and GSI AudioStar Pro diagnostic audiometers. Air-conduction (AC) thresholds were obtained at 125, 250, 500, 1,000, 2000, 4,000, and 8,000 Hz using TDH-39 headphones. Bone-conduction (BC) thresholds were measured at 250, 500, 1,000, 2000, and 4,000 Hz using a B71 bone vibrator. The preoperative AC pure-tone average (PTA) at 500, 1000, 2000, and 4,000 Hz and the corresponding preoperative ABG (AC-BC) at these frequencies were calculated from the last preoperative audiogram. Postoperative PTA and ABG at the same frequencies were calculated from the follow-up audiogram.

### 2.4 Statistical analysis

Data were analyzed using SPSS version 26.0. The distributions of WBA, RF, and ABG were assessed for normality using the Shapiro–Wilk test. For variables that met the assumption of normality (*p* > 0.05), independent-samples t-tests were used to compare postoperative WBA and RF between the study and control groups. Paired t-tests were used to compare pre- and postoperative ABG within the study group. Spearman’s rank correlation analysis was performed to examine the relationships between WBA, RF, and ABG. In addition, postoperative WBA at 1000 Hz was compared between temporalis fascia and tragal perichondrium graft subgroups using independent-samples t-tests. A two-sided *p* value < 0.05 was considered statistically significant.

## 3 Results

### 3.1 Subject characteristics

Forty-four patients with unilateral chronic otitis media who underwent endoscopic type I tympanoplasty and had a normal contralateral ear were included in the analysis (44 operated ears and 44 contralateral ears). The overall mean age was 48.34 ± 12.23 years (17 males: 49.35 ± 14.33 years; 27 females: 47.70 ± 10.95 years). The mean duration of disease before surgery was 5.05 ± 7.49 years (median, 1.88 years). The mean interval between surgery and WBT measurement was 1.24 ± 0.63 years.

### 3.2 Comparison of WBA between groups

**Figure 1:**
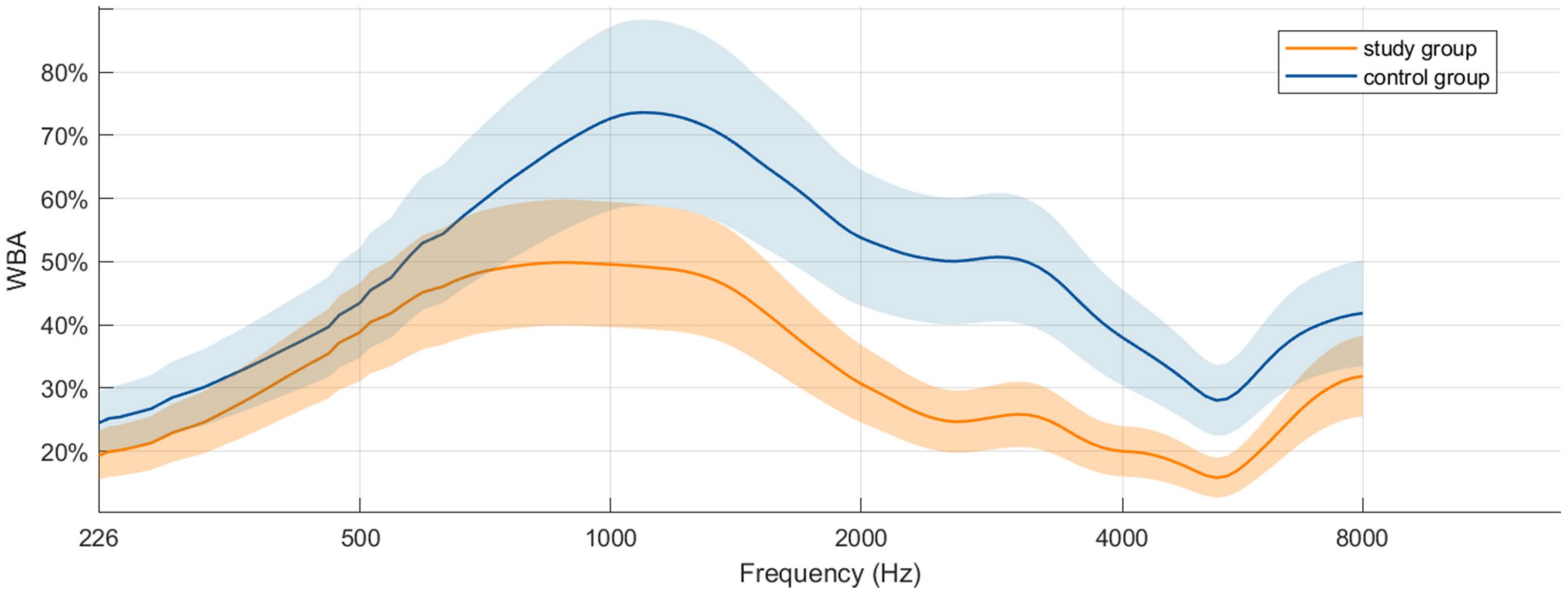
Mean WBA curves for the Study Group and Control Group. The shaded area represents the 95% confidence interval (CI).

The WBA values across the 107 test frequencies were plotted as mean absorbance curves for the study and control groups. In both groups, the frequency-WBA curves showed a broadly similar asymmetric M-shaped configuration (Figure 1). In the study group, WBA peaks occurred at 890 Hz (0.50 ± 0.16) and 3,084 Hz (0.26 ± 0.21), whereas in the control group the corresponding peaks were at 1090 Hz (0.74 ± 0.15) and 2,911 Hz (0.51 ± 0.21). Across the tested frequency range, mean WBA was lower in the study group, with a broader, flatter first peak (approximately 840–917 Hz), a main trough at 5339 Hz, and overall values remaining below 50%.

**Figure 2:**
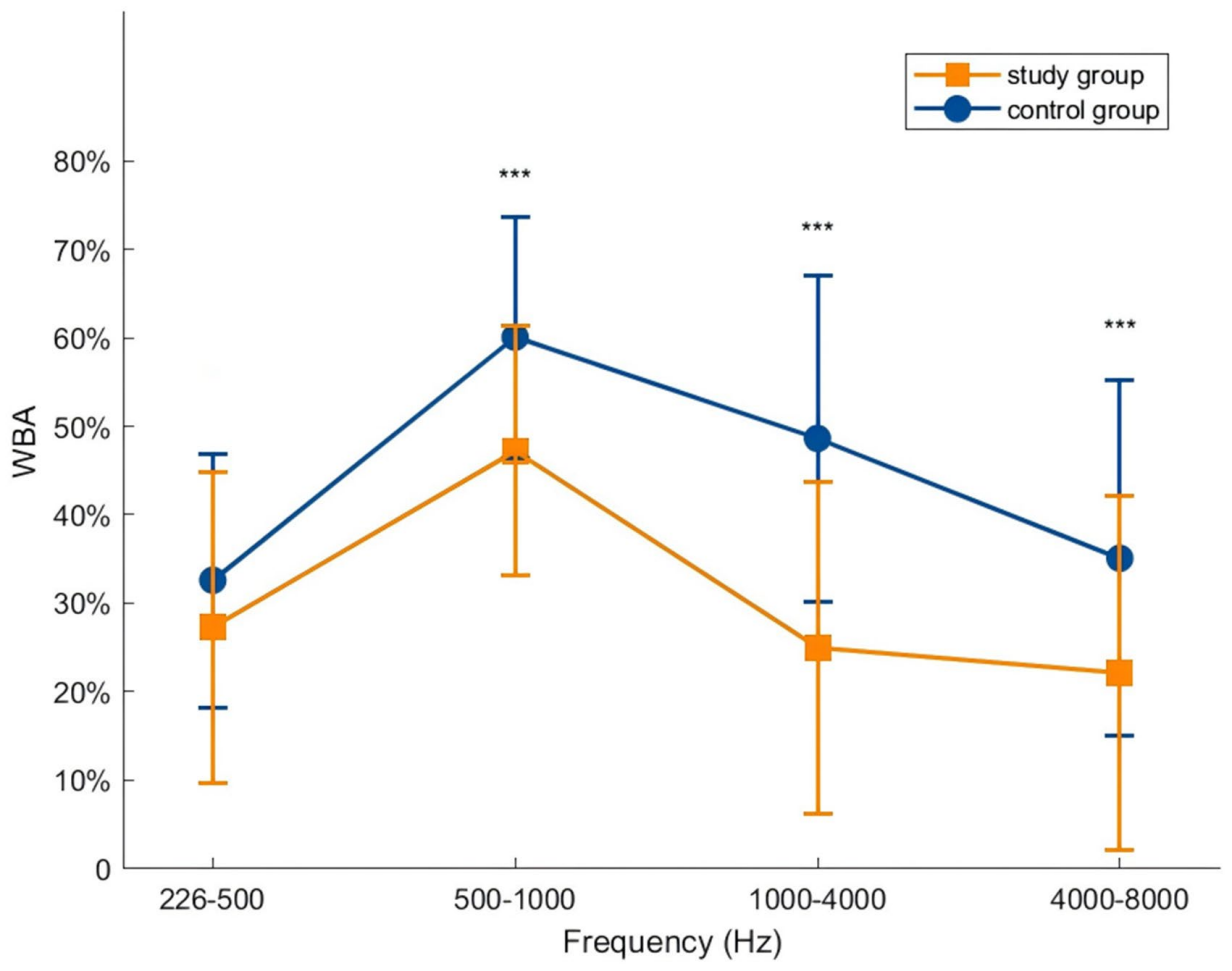
Comparison of WBA between the Study Group and Control Group across four frequency bands (mean ± SD). Error bars represent standard deviation (SD) (*** *p* < 0.001).

For statistical analysis, the 107 test frequencies were grouped into four bands: low (226–500 Hz), mid-low (500–1,000 Hz), mid-high (1000–4,000 Hz), and high (4000–8,000 Hz). Independent-samples t-tests showed statistically significant differences in mean WBA between the study and control groups in the mid-low, mid-high, and high frequency bands (*t* = −4.068/−5.969/−3.875, all *p* < 0.001). In the low-frequency band (226–500 Hz), mean WBA tended to be lower in the study group but did not reach statistical significance (*t* = −1.909, *p* > 0.05). The differences were most pronounced in the mid-high frequency band (1000–4,000 Hz), with a maximal between-group difference of 25.5% at 2669–2747 Hz, and smallest in the low-frequency band (Figure 2).

### 3.3 Comparison of middle-ear RF between groups

**Figure 3:**
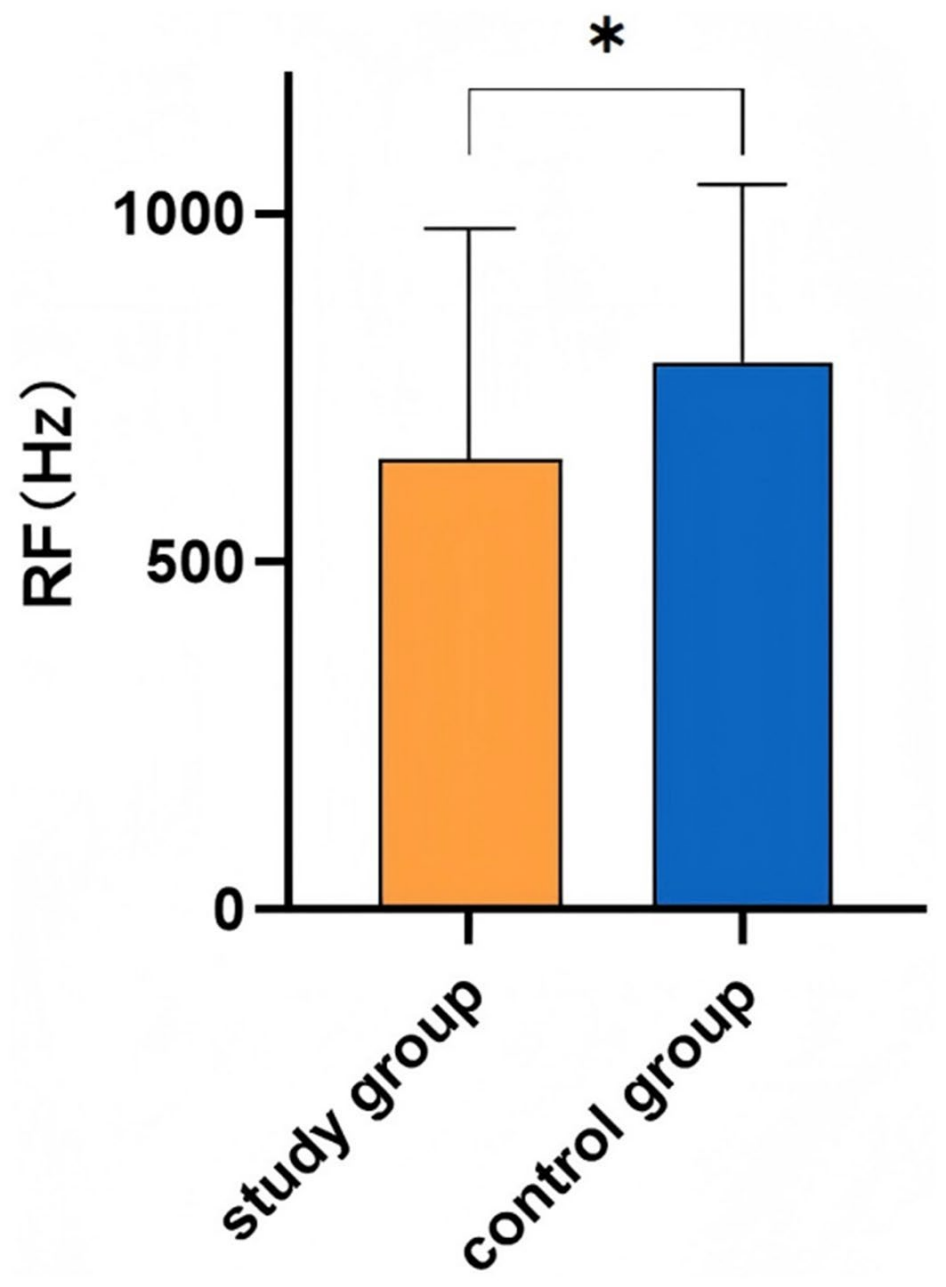
Comparison of Middle-Ear RF between the Study Group and Control Group (mean ± SD). Error bars represent SD (* *p* < 0.05).

The mean middle-ear RF in the study group (648.95 ± 329.73 Hz) was significantly lower than that in the control group (787.31 ± 254.95 Hz) (*t* = −2.225, *p* < 0.05; Figure 3).

### 3.4 Hearing recovery in the study group (pre- vs. postoperative)

**Figure 4:**
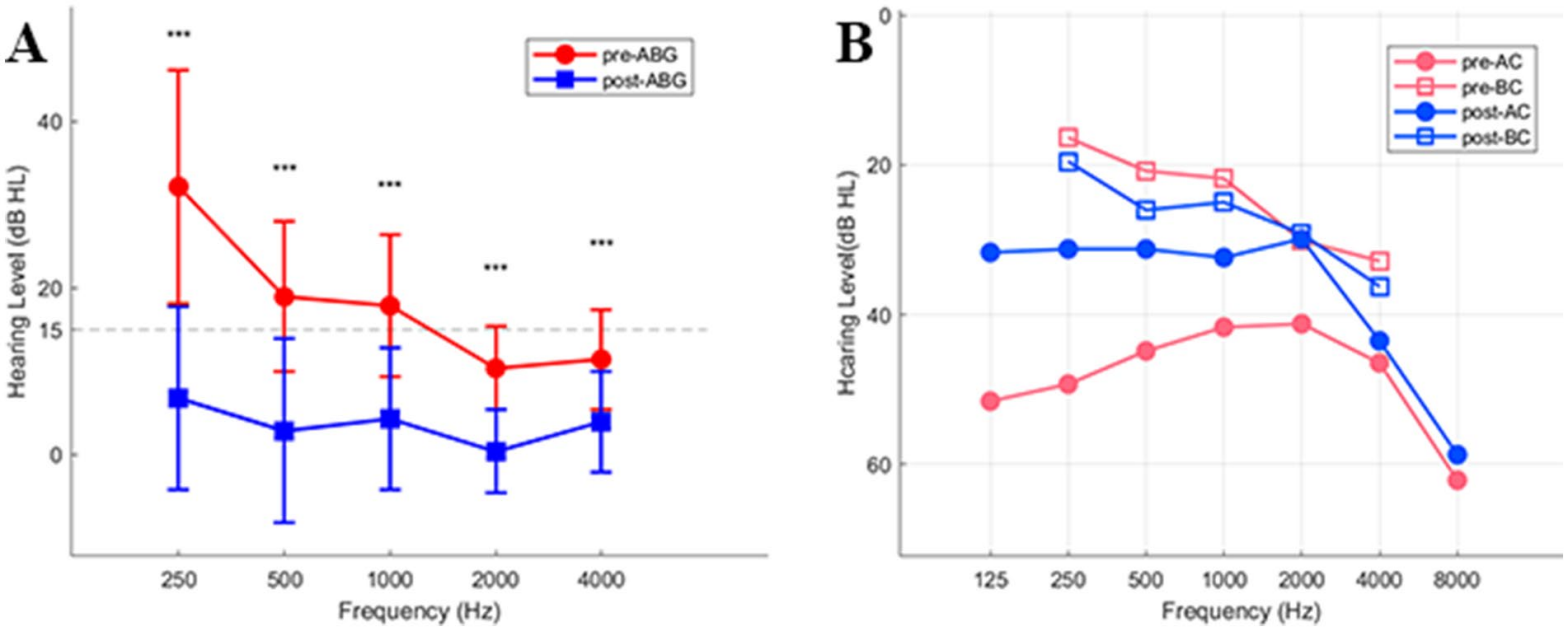
Pre- and post-operative hearing recovery in the Study group. **(A)** Comparison of pre- and postoperative ABG across test frequencies (mean ± SD). Error bars represent SD. **(B)** Comparison of pre- and postoperative AC and BC thresholds (*** *p* < 0.001). Pre-AC: preoperative air conduction; Pre-BC: preoperative bone conduction; Post-AC: postoperative air conduction; Post-BC: postoperative bone conduction.

Comparison of pre- and postoperative pure-tone thresholds in the operated ears showed significant improvement in AC thresholds, particularly at the low frequencies, whereas BC thresholds did not change significantly (all *p* > 0.05; Figure 4A). Paired t-tests showed that postoperative ABG was significantly reduced compared to preoperative ABG at 250, 500, 1,000, 2000, and 4,000 Hz (*t* = 10.867/9.832/7.319/ 6.711/4.206, all *p* < 0.001). The mean postoperative ABG at all tested frequencies was less than 15 dB HL (Figure 4B).

### 3.5 Correlation between postoperative ABG, RF, and WBA

**Figure 5:**
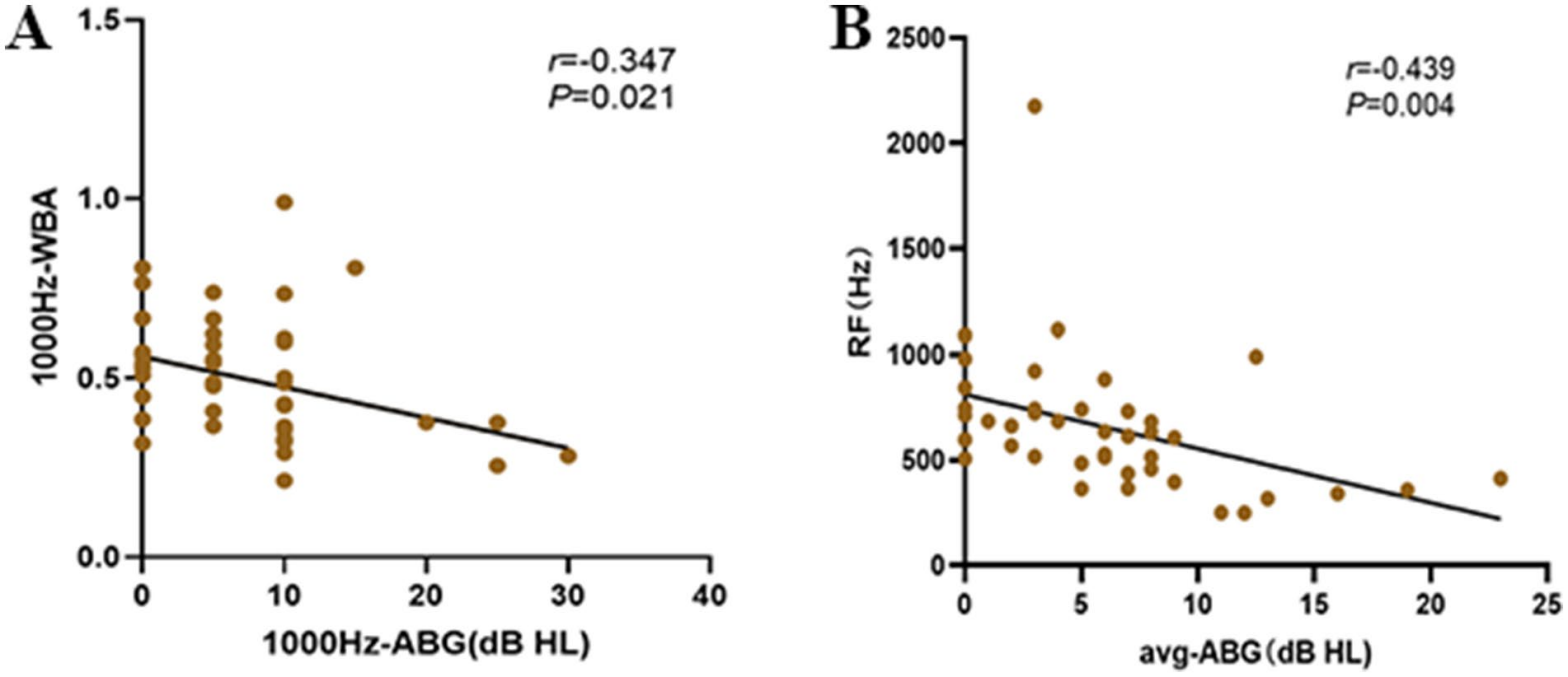
Postoperative correlations between RF, WBA, and ABG in the Study group. **(A)** Correlation between postoperative ABG and WBA at 1000 Hz. **(B)** Correlation between postoperative ABG and RF.

Spearman’s rank correlation analysis in the study group showed a significant negative correlation between postoperative ABG and WBA at 1000 Hz (*r* = −0.347, *p* < 0.05), whereas correlations at 250, 500, 2000, and 4,000 Hz were not statistically significant (all *p* > 0.05; Table 1; Figure 5A). Analysis showed a significant negative correlation between mean postoperative ABG and RF (*r* = −0.439, *p* < 0.05), indicating that smaller ABG values were associated with higher RF values (Figure 5B).

**Table 1:** Correlation coefficients between WBA and ABG in the study group.

Frequency (Hz)	WBA (Mean ± SD)	ABG (dB HL, Mean ± SD)	*r*	*p*
250	0.20 ± 0.07	11.14 ± 8.75	−0.041	0.792
500	0.39 ± 0.13	5.34 ± 8.03	−0.204	0.878
1,000	0.50 ± 0.17	7.61 ± 7.03	−0.347	0.021
2000	0.31 ± 0.22	1.02 ± 2.55	−0.024	0.876
4,000	0.20 ± 0.22	6.63 ± 6.96	−0.041	0.796

The study group was further subdivided into a temporalis fascia graft subgroup (*n* = 23) and a tragal perichondrium graft subgroup (*n* = 21). Independent-samples t-tests showed no significant difference in WBA at 1000 Hz between the two groups (*t* = −0.125, *p >* 0.05).

## 4 Discussion

The primary objective of type I tympanoplasty is functional restoration of the middle ear, which fundamentally depends on the integrity and physiological competence of the tympanic membrane (7). Transmission of acoustic energy through the middle ear is determined by its biomechanical properties, including mass, stiffness, and frictional resistance. After surgical repair, the grafted tympanic membrane may exhibit altered sound energy absorption because of changes in its mass and stiffness characteristics. WBT provides a comprehensive assessment of the middle ear’s acoustic response over a broad frequency spectrum and markedly improves the detection of subtle pathophysiological changes. Quantitative parameters such as WBA and RF offer detailed insight into the mechanical behavior of the middle ear and serve as valuable objective indices for postoperative evaluation.

### 4.1 WBT characteristics after endoscopic type I tympanoplasty

In the present study, the frequency-absorbance curves for both the operated and contralateral normal ears demonstrated an asymmetric M-shaped configuration with a more prominent first peak. Mazlan et al. (9) plotted wideband absorbance curves in normal young adults and also observed an M-shaped pattern; however, in their study the second peak was higher than the first. This differs from the findings in our control group and may be related to the older mean age of our cohort (approximately 48 years). In some patients, mild tympanic membrane or ossicular chain calcification associated with aging may increase the effective mass of the system and consequently reduce high-frequency absorbance (10). In our study, the postoperative peak was broader and flatter than that of the contralateral ear and occurred at 890 Hz. Asta et al. (11) investigated WBA at ambient pressure after type I tympanoplasty and reported a peak WBA of 0.58 at 1000 Hz at 3 months postoperatively, which was also broader and flatter than preoperatively, consistent with our observations. Karuppannan et al. (12) reported significantly reduced WBA across the frequency range in ears with middle-ear effusion and reduced WBA below 2,500 Hz in ears with TMP. Xu et al. (13) found a WBA peak of 0.56 around 1,100 Hz in chronic suppurative otitis media, whereas cholesteatoma showed a lower peak (0.34) around 710 Hz. These findings suggests that peak frequency and peak WBA may help differentiate among middle-ear pathologies. Our postoperative WBA curve can therefore be interpreted in relation to normal patterns and those observed in other disease entities, thereby enriching diagnostic differentiation.

Previous studies have suggested that grafted tympanic membranes may lack sufficient elasticity after repair because of incomplete remodeling or inadequate mechanical support, which can lead to retraction and impaired sound conduction (14, 15). Furthermore, insufficient preservation or reconstruction of tension-related anatomical structures (e.g., the malleus handle and tensor tympani tendon) during surgery may directly affect postoperative membrane mechanics (16). The reduced absorbance observed in our study group is therefore likely to reflect decreased membrane tension and altered mechanical properties of the grafted tympanic membrane compared with the native membrane.

In the present study, differences in WBA between operated and normal ears were small at 226–500 Hz and did not reach statistical significance, but increased progressively at frequencies above 500 Hz, with the largest discrepancies occurring in the 1,000–4,000 Hz range. Nesibe et al. (17) reported similar WBA below 1 kHz between normal and grafted tympanic membranes, but lower WBA above 1 kHz in grafted ears. Eberhard et al. (18) found lower WBA in fascia-grafted ears than in normal tympanic membranes, particularly at mid-to-high frequencies, indicating reduced sound energy transmission. Asta et al. (11) observed comparable low-frequency WBA between postoperative ears (3 months, ambient pressure) and healthy controls, but significantly lower WBA above 771 Hz in the postoperative group. These findings are consistent with our results. In addition to reduced graft elasticity, increased graft thickness and mass may further limit sound conduction. High-frequency sound transmission is primarily mass controlled, and the increased effective mass of the repaired tympanic membrane relative to the physiological state likely contributes to the persistently lower high-frequency WBA observed postoperatively.

RF is defined as the frequency at which the stiffness and mass susceptances of the middle ear are equal in magnitude and opposite in sign. Accordingly, changes in ossicular chain stiffness or mass will result in shifts in RF. RF has been widely used in the diagnosis of middle-ear disease and has shown high sensitivity for subtle pathologies involving the tympano-ossicular system (19), such as in the assessment of otitis media with effusion and the diagnosis of otosclerosis. It has also been applied to the evaluation of inner-ear disorders (20, 21), including Ménière’s disease and large vestibular aqueduct syndrome. In our study, the lower mean RF in the study group suggests reduced tympanic membrane stiffness and increased mass after surgery. The negative correlation between RF and postoperative ABG indicates that higher RF values, reflecting mechanics closer to normal, are associated with smaller ABG values and thus better hearing outcome, suggesting that RF may serve as an additional parameter for postoperative outcome assessment.

### 4.2 Correlation between WBA and hearing recovery

Clinical studies have reported high perforation closure rates and favorable hearing recovery after type I tympanoplasty (6, 22), with most patients achieving a mean postoperative ABG < 15 dB (23–25). In our cohort, postoperative ABG was significantly reduced compared with preoperative ABG, consistent with these findings and indicating satisfactory hearing recovery. However, only minimal improvement in AC thresholds was observed at 4000–8000 Hz. This may be attributable to differences in the structural and mechanical properties of the graft material compared with the native tympanic membrane (26), which can adversely affect high-frequency sound transmission. Park et al. (27) found a significant correlation between postoperative ABG and WBA at 1000 Hz measured at 6 and 12 months after surgery in patients with chronic otitis media. Similarly, we identified a negative correlation between WBA and ABG at 1000 Hz in the operated ears. This suggests that higher WBA at 1000 Hz is associated with better ABG closure and hearing recovery, and that WBA at this frequency may serve as a potential clinical indicator of postoperative hearing outcome.

We further analyzed WBA at 1000 Hz according to the type of graft material and found no significant difference between temporalis fascia and tragal perichondrium grafts. This finding suggests that, under current surgical practice, the two graft materials perform comparably with respect to postoperative WBA at 1000 Hz. Nevertheless, postoperative WBA in grafted ears remained generally lower than in contralateral normal ears. It is therefore necessary to continue exploring graft materials and surgical techniques that more closely mimic the mechanical properties of the native tympanic membrane, in order to optimize postoperative middle-ear mechanics and provide stronger evidence for clinical decision-making.

## 5 Limitations

This retrospective study included subjects with a wide range of ages and disease durations, which may have contributed to variability in WBA across frequencies. In addition, the relatively small sample size warrants future studies with larger cohorts to allow a more comprehensive analysis of postoperative WBT characteristics. Some patients also presented with bilateral symmetrical high-frequency sensorineural hearing loss, which may not fully reflect the postoperative status of isolated unilateral chronic otitis media after tympanoplasty. More stringent patient selection and further stratified analyses are therefore needed in future studies.

## 6 Conclusion

WBT confirmed significant alterations in middle-ear sound energy transmission after endoscopic type I tympanoplasty. Overall, WBA across the tested frequency range was lower in operated ears than in contralateral normal ears, with statistically significant differences in the mid-low, mid-high, and high frequency bands and the largest discrepancies in the 1,000–4,000 Hz range. The absorbance peak in the operated ears was broader, flatter, and shifted toward lower frequencies, and RF was concurrently decreased, indicating increased tympanic membrane mass and reduced stiffness. A key finding of this study was the significant negative correlation between WBA at 1000 Hz and postoperative ABG, suggesting that absorbance at this frequency may serve as an objective predictor of the degree of hearing recovery. Taken together, WBT parameters, particularly WBA and RF, provide objective quantitative tools for postoperative assessment of middle-ear function, and WBA at 1000 Hz appears to have prognostic value for postoperative hearing outcomes.

## Data Availability

The raw data supporting the conclusions of this article will be made available by the authors, without undue reservation.
